# Clinical Profile and Predictors of Survival in Carcinoma Penis Patients

**DOI:** 10.3390/curroncol30050345

**Published:** 2023-04-28

**Authors:** Vikas Garg, Mukurdipi Ray, K. P. Haresh, Ranjit Kumar Sahoo, Atul Sharma, Seema Kaushal, Atul Batra

**Affiliations:** 1 Department of Medical Oncology, BR Ambedkar Institute Rotary Cancer Hospital, All India Institute of Medical Sciences, Delhi 110029, India; 2 Department of Surgical Oncology, BR Ambedkar Institute Rotary Cancer Hospital, All India Institute of Medical Sciences, Delhi 110029, India; 3 Department of Radiation Oncology, BR Ambedkar Institute Rotary Cancer Hospital, All India Institute of Medical Sciences, Delhi 110029, India; 4 Department of Pathology, All India Institute of Medical Sciences, Delhi 110029, India

**Keywords:** carcinoma penis, penile cancer, chemotherapy, prognosis, predictors, education, rural, urban, overall survival

## Abstract

Background: Carcinoma penis is a rare neoplasm, and the literature is scarce on long-term survival and its predictors. The aim of the study was to determine the clinical profile and management patterns, identify predictors of survival, and the impact of education and rural/urban dwelling on survival. Methods: Patients with a histological diagnosis of carcinoma penis from January 2015 to December 2019 were included in the study. Demographics, clinical profile, education status, primary residence address, and outcomes were obtained from the case records. Distance from the treatment centre was obtained from the postal code. The primary objectives were to assess relapse-free survival (RFS) and overall survival (OS). The secondary objectives were to identify the predictors of RFS and OS and to determine the clinical profile and treatment patterns in patients with carcinoma penis in India. Time-to-event was calculated by Kaplan–Meir analysis and survival was compared by the log-rank test. Univariate and multivariable Cox regression analyses were used to find independent predictors of relapse and mortality. Logistic regression analyses to examine the associations of rural residence, education status, and distance from the treatment centre with the relapse adjusting for measured confounding variables. Results: Case records of 102 patients treated during the above period were retrieved. The median age was 55.5 (interquartile range [IQR] 42–65 years). Ulcero-proliferative growth (65%), pain (57%), and dysuria (36%) were the most common presenting features. Clinical examination or imaging revealed inguinal lymphadenopathy in 70.6% of patients, however, only 42% of these lesions were pathologically involved. A total of 58.8% of patients were from rural areas, 46.9% had no formal education, and 50.9% had a primary residence ≥100 km from the hospital. Patients with lower education and rural households had higher TNM stages and nodal involvement. Median RFS and OS were 57.6 months (15.8 months to not reached) and 83.9 months (32.5 months to not reached), respectively. On univariate analysis tumor stage, involvement of lymph nodes, T stage, performance status, and albumin was predictive for relapse and survival. However, on multivariate analysis, the stage remained the only predictor of RFS and nodal involvement, and metastatic disease was a predictor of OS. Education status, rural habitation, and distance from the treatment centre were not predictors for relapse or survival. Conclusions: Patients with carcinoma have locally advanced disease at presentation. Rural dwellings and lower education were associated with the advanced stage but did not have a significant bearing on the survival outcomes. The stage at diagnosis and nodal involvement is the most important predictor of RFS and OS.

## 1. Introduction

Penile cancer is an uncommon malignancy of the genitourinary system accounting for less than one percent of adult malignancies in developed countries [1]. However, it is relatively common in the developing world and may account for up to 10% of all cancers in men in some African and South American countries [2,3]. In India, the estimated incidence rate is 0.8 per 100,000 according to the recent population-based cancer registry report [4].

It is a disease of elderly men with a median age of 60 years and usually presents as an ulcero-proliferative growth over the penis [5,6]. It spreads via lymphatics to involve inguinal lymph nodes and subsequently, pelvic and retroperitoneal lymph nodes. Inguinal lymph node involvement is common at presentation (30–60%), however distant metastasis is rare at diagnosis (<10%) [7].

Squamous cell carcinoma is the most prevalent subtype, accounting for approximately 95% of all cases. Less common varieties include papillary, condylomatous, basaloid, verrucous, and sarcomatoid carcinoma [8]. Risk factors for the development of carcinoma penis include genital warts, poor hygiene, urinary tract infections, penile tear, penile injury, phimosis, human immunodeficiency virus (HIV) infection, and smoking [9,10]. Infection with the human papilloma virus (HPV), particularly types 16 and 18, which can be identified in up to 50% of squamous cell carcinoma patients, is frequently linked to favourable outcomes [11,12,13]. Tumor stage, grade, lymph node involvement, Ki-67, and HPV status are considered the most important prognostic factors [14,15].

The primary treatment for penile cancer is surgical, although in very low-risk cases organ preserving therapies like laser therapy or brachytherapy may be employed [16,17,18]. Lymph node staging is an important part of management and may be done by either fine needle aspiration cytology (FNAC), superficial inguinal lymph node dissection (ILND), or dynamic sentinel node biopsy (DSNB) [19]. The role of neoadjuvant chemotherapy is limited to patients presenting with unresectable primary, bulky, or bilateral inguinal lymph nodes, or pre-operative imaging suggestive of pelvic lymphadenopathy [20,21,22]. Adjuvant chemotherapy is indicated in the presence of bilateral or multiple inguinal lymph nodes, extranodal extension (ENE), or pelvic lymph node involvement [23]. Metastatic disease is treated with palliative intent with platinum-based chemotherapy [24,25].

The literature on the clinical profile and treatment outcomes in Indian patients with carcinoma penis is scarce. The impact of education status, rural versus urban dwelling, and distance from the treatment centre has not been reported in India. The objective of this study was to determine the clinical profile, management patterns, and treatment outcomes in patients with carcinoma penis. Further, we identified factors that predict survival and studied the impact of education, rural habitation, and distance from a treatment centre. Understanding the profile and predictors of survival in these patients can help devise strategies to better prognosticate and manage patients effectively.

## 2. Materials and Methods

### Methodology

This retrospective study was conducted at the Department of Medical Oncology of a tertiary care centre in India after approval from the institute’s ethics review board. Patients ≥18 years of age with a histological diagnosis of carcinoma penis from January 2015 to December 2019 were included in the study. Baseline demographics profile, education status, primary residence address, presenting symptoms, staging, histopathologic features, detailed treatment summary, and response to treatment, were obtained from case records. Management including treatment modality (surgery, radiation therapy, chemotherapy, or combination) and intent of management viz. curative, or palliative was also recorded. Palliative therapy was used to describe any treatment modality administered in cases where a cure was not anticipated. The staging was done according to the American Joint Committee on Cancer (AJCC) tumor, node, metastasis (TNM) seventh edition staging system [26]. Distance from the treatment centre was obtained from the postal code. The rural area was denoted as per the census of India 2011, which has a population of less than 5000, a density of population of less than 400 per square km, and more than 25 percent of the male working population is engaged in agricultural pursuits [27]. The date of diagnosis, date of relapse, duration between diagnosis to death, or last follow-up were also recorded.

The primary objectives were to assess the relapse-free survival (RFS) and overall survival (OS) in patients with carcinoma penis. The secondary objectives were to identify the predictors of RFS and OS and to determine the clinical and demographic profile and treatment patterns in patients with carcinoma penis in India. Relapse-free survival was defined as the period from the date of initial surgery or other curative treatment to the date of relapse. Overall survival (OS) was defined as the period from the date of diagnosis to death due to any cause. Data were censored at the most recent follow-up.

## 3. Statistical Analysis

Data were analyzed by Stata 14 (4905 Lakeway Drive, College Station, TX, USA) and presented in mean ± standard deviation (SD)/median (range) and frequency (percentage). Categorical variables were compared by Chi-square or Fisher’s exact test. Continuous variables following normal distribution were compared by independent *t*-test. Data not following normal distribution was compared by the Wilcoxon Rank sum test. Time-to-event was calculated by Kaplan–Meir analysis and survival was compared using the log-rank test. Univariate and multivariable Cox regression analysis was used to find independent predictors of survival and mortality. Adjusted/unadjusted death or last follow-up was used for calculating the hazard ratio. A *p*-value <0.05 was considered statistically significant.

## 4. Results

### Demographic Data

A total of 102 patients with histological diagnoses of carcinoma penis were treated from January 2015 to December 2019. The baseline clinical characteristics of patients with carcinoma penis are summarized in Table 1. The median age of the population was 55.5 years (interquartile range [IQR] 42–65 years) and the median duration from symptom onset to diagnosis was 6.0 months (IQR, 4–12 months). Smoking was the most common (27.5%) risk factor associated with carcinoma penis followed by phimosis (25.5%), balanoposthitis (21.6%), and HIV (1.9%). A history of circumcision was present in only one patient, and it was performed just after birth.

The most common presenting symptoms were pain followed by discharge from the lesion and urinary symptoms. About 30% of patients had an ECOG (Eastern Cooperative Oncology Group) performance status score of 2 or more at the time of presentation. Glans penis (70.6%) was the most involved site followed by the shaft of the penis (29.4%). In 65.7% of cases, the primary lesion presented as an ulcero-proliferative growth, while the rest presented either as an ulcer or a lump on the penis. On examination, inguinal lymph node involvement and scrotal involvement were observed in 71.6% and 7.8% of patients, respectively.

## 5. Histopathologic Profile

Baseline tumor characteristics including TNM staging and histopathologic findings have been summarized in Table 2. The most common T stage at presentation was T3 in 36.2% of patients. Pathologic involvement of lymph nodes at diagnosis was present in 42.1% of patients. Distant metastases at diagnosis were observed only in 3.8%. A majority (44.1%) had localized disease (stage I & II), while advanced or locally advanced disease was observed in 43.1%. Complete staging status was not available in 12.8% of cases. Status on lymphovascular invasion (LVI) and perineural invasion (PNI) were available only in about 40% of cases. The most common histology was moderately differentiated squamous cell carcinoma in 60.8% of cases. Rare histology (adenocarcinoma, basaloid, sarcomatoid) was observed in only 2.9% of patients.

## 6. Impact of Education, Rural Household, and Distance from Treatment Centre

Most of the patients (58.8%, *n* = 62) belonged to rural households and about half of the patients (50.9%, *n* = 52) had to travel ≥100 km for accessing cancer care. The patient had low education attainment with less than one-third (31.3%) having schooling at more than primary school, while 46.9% had received no formal education at all. Among patients with node-positive disease (*n* = 43), 29 (67.4%) patients attended primary school or had no formal education, and 30 (69.7%) patients belonged to rural areas. Similarly, among patients with stage III or IV disease (*n* = 44), 30 (68.1%) patients attended primary school or had no formal education, and 32 (72.7%) patients belonged to rural areas. Differences in baseline characteristics based on education, rural household, and distance from the treatment centre have been elaborated in Table 3.

There was no significant difference in age, presence of risk factors (smoking, phimosis), ECOG PS, and duration of symptoms preceding diagnosis of carcinoma penis. Patients belonging to the rural household, residing >100 km away from the treatment centre, and those with lower educational attainment were more likely to have adverse tumor characteristics viz. higher T stage, node positivity, advanced stage at diagnosis, and presence of distance metastasis. However, the difference did not reach significance except for the T stage (based on distance from the treatment centre) and stage at the time of diagnosis (based on education).

## 7. Management

About 44% of patients had received some form of treatment before presentation to our centre. Details of previous treatment and relapse status at presentation to our institute are summarized in Supplementary Table S1. Of these patients, all patients except one (who received palliative chemotherapy) underwent initial surgical management. Partial penectomy was performed in two third of patients (66.6%) while the remaining underwent either circumcision (21.8%) or total penectomy (4.4%). Inguinal lymph node dissection (ILND) was performed in 8.8% and adjuvant radiotherapy was administered in 4.4% of patients, respectively. None of the patients received adjuvant chemotherapy. Post initial management 17.8% had a local relapse, 22.2% of patients had relapsed at distant (+/−local) sites, while 57.7% had no relapse.

The initial management of patients at our centre is summarized in Supplementary Table S2. The majority (76.8%) of patients underwent initial surgery, of which concomitant ILND was performed in 51.1% (*n* = 22) patients while the rest had only SLNB or ILND later. Curative intent brachytherapy with external beam radiation therapy (EBRT) was delivered in 3.6% (*n* = 2). Among patients undergoing surgery, 88.4% had organ preservation surgery (partial penectomy) and only 11.6% of patients underwent total penectomy. Patients belonging to rural areas (66.6%), residing farther from the specialized centre (66.6%), and low education level (83.3%) were more likely to undergo total penectomy. Palliative chemotherapy or radiotherapy was started in 17.8% of patients and one patient (1.8%) was advised best supportive care (BSC).

Overall chemotherapy was administered in 24 patients in adjuvant (*n* = 6) or palliative settings (*n* = 18). Six patients were started on adjuvant chemotherapy either TIP (paclitaxel, ifosfamide, and cisplatin) or TP (paclitaxel and carboplatin). Palliative chemotherapy consisted of either TP/TIP in 66.6% (*n* = 12) patients, methotrexate with/without TKI (Tyrosine kinase inhibitors) in 22.2% (*n* = 4), or TKI alone in 11.1% (*n* = 2). Gefitinib or erlotinib was used in 11.1% (*n* = 2) of patients with poor performance status. The objective response rate (ORR) to palliative systemic therapy was 50%. Responses were observed in three out of four patients on TKI. Radiotherapy was administered in 39 patients including six patients receiving definitive radiotherapy, adjuvant in 19 patients, and 14 in a palliative setting.

## 8. Survival

**Figure 1:**
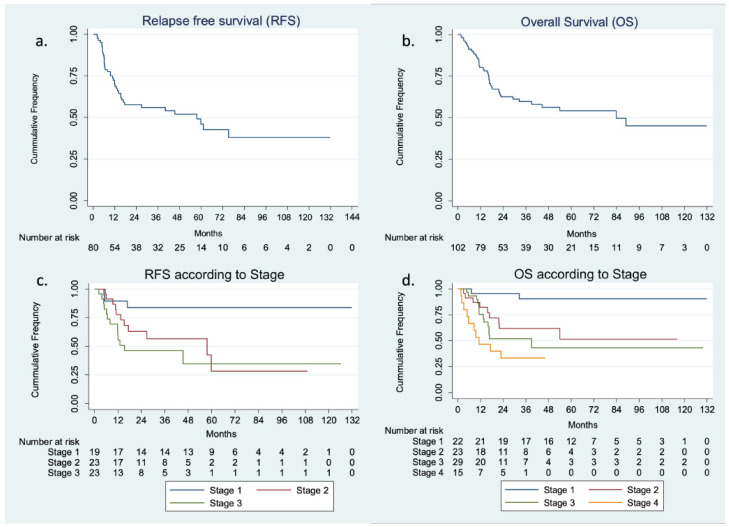
Kaplan–Meir estimates of (**a**). Relapse-free survival (RFS) in total population (**b**). Relapse-free survival (RFS) is according to stage (**c**). Overall survival (OS) in total population (**d**). Overall survival (OS) according to the stage.

Of 80 patients treated with curative intent, 40 patients had relapsed at the last follow-up. Median relapse-free survival (RFS) was 57.6 months (15.8 months not reached [NR]) (Figure 1). On univariate (Table 4) analysis poor performance status, low serum albumin, higher t-stage, nodal involvement, and advanced stage were significant predictors for relapse. Education status, rural versus urban dwelling, and distance from the hospital were not predictive of relapse. However, on multivariate analysis, stage at presentation (hazard ratio [HR] 1.75; 95% confidence interval [CI] 1.07 to 2.85; *p* value 0.02) remained statistically significant predictors for relapse.

At the last follow-up, 43 patients out of a total of 102 had died. Median overall survival (OS) was 83.9 months (32.5 months to NR) (Figure 1). On univariate analysis poor performance status, low serum albumin, presence of PNI, higher T stage, nodal involvement, metastatic disease, and advanced stage were significant predictors for death. Education status, rural versus urban dwelling, and distance from the hospital were not predictive. However, on multivariate analysis, nodal involvement (HR 3.58; 95% CI 1.52 to 8.41; *p* value 0.003), and metastatic disease (HR 3.55; 95% CI 1.02 to 12.34; *p* value 0.045) remained statistically significant predictors. Median survival for stage I and stage II were not reached, while for stage III and stage IV were 39.2 months (13.8 months not reached) and 11.2 months (3.2 months not reached), respectively.

There was no impact of distance, education, or rural/urban dwelling on RFS and OS in carcinoma penis patients. Univariate and multivariate analysis (Table 4) suggest that none of these factors predicted RFS and OS.

## 9. Discussion

Penile cancer poses a considerable social and economic burden in developing nations [28]. In this study, health records of 102 patients with carcinoma penis treated at a tertiary care centre in India were analyzed to determine the clinical profile, treatment patterns, outcomes, and predictors.

The median age in our study was 55 years which is similar to that reported in other studies from India [29,30]. Penile carcinoma has a median age of around 60 years in Western countries and a decade younger in developing nations [31,32,33]. Smoking (27.5%), phimosis (25.5%), balanoposthitis (21.6%), and HIV (1.9%) were commonly observed risk factors in this study. Poor hygiene, genital warts, infections, phimosis, HIV, HPV, and smoking are known risk factors, while circumcision at birth is protective. In our series, only one patient had a prior history of circumcision at birth [9,10].

Carcinoma penis most commonly presents as a painless lump or ulcer, however more than half of patients in the current study presented with painful ulcer-proliferative growth [5]. Discharge and dysuria were also complained of by one-third of patients. Similarly, clinically inguinal lymphadenopathy (70%) and scrotal involvement (8%) were observed in a high proportion of patients than reported in the literature [7]. Patients had higher T stage (only 25% ≤ T2) and N stage (N+ in 42.1%) as reported from various developing nations [33,34]. Overall, 43.1% of patients had either stage III or IV at presentation. This may be due to more advanced diseases at presentation due to delays in healthcare access.

Well or moderately differentiated carcinoma was observed in about 90% of patients, while poorly differentiated and rare histology was reported in <10%. This is similarly reported in other studies [29,30]. LVI or PNI was reported in only 40% of patients and was not available for the rest.

About 80% of patients were treated with curative intent with initial surgery. Patients with localized disease underwent penectomy with/without inguinal lymph node dissection. However, most patients treated at non-specialized centres did not undergo the required inguinal lymph node staging or organ preservation approaches due to a lack of facilities [35,36]. A study by the United States (US) National Cancer Database reported that only 27.2% of patients with carcinoma penis underwent inguinal lymphadenectomy. Further, the procedure was associated with increased OS. Most patients treated at our centre had lymph node staging in form of SLNB or ILND.

Palliative therapy was initiated in about 20% of patients with the majority receiving platinum-based therapy [31]. Patients with poor PS were started on TKIs with or without methotrexate. Although the numbers are small, good responses (three out of four) were observed with TKI-based therapy in a palliative setting. Other EGFR inhibitors have also shown some promise but these are all in retrospective series [37,38].

Median OS for stage I and stage II were not reached, while for stage III and stage IV it was 39.2 months and 11.2 months, respectively. Advanced stage, higher T stage, nodal involvement, metastatic disease, poor ECOG PS at diagnosis, low serum albumin, and presence of PNI, were significant predictors for death. However, the stage was not a significant predictor for OS on multivariate analysis and only nodal involvement, and metastatic disease retained significance. Various predictive factors for OS have been identified but nodal involvement is the most consistent [38,39,40,41,42,43,44].

Most of the patients belonged to rural areas, had some or no formal education, and resided far from the tertiary centre. Due to this access to appropriate healthcare may be challenging and most patients have locally advanced disease when they are diagnosed [33,34]. Rural habitation and low education were associated with higher T stage, higher N stage as well as Stage III/IV. Similarly, patients belonging to rural areas and with lower educational attainment had higher chances of undergoing suboptimal initial surgery or radical penectomy. However, there was no impact of these factors on RFS or OS in this study. Studies have identified rural habitation as a predictor of higher stage and poor outcomes [45,46]. Similarly, lower education is also associated with delayed diagnosis, higher disease stage, inferior outcomes, and a higher likelihood of radical penectomy [44,45,46,47].

In a single registry-based study from an urban population in India, the impact of education, religion, marital status, and age was evaluated in patients diagnosed with carcinoma cervix and carcinoma penis. Illiterate patients had double the incidence of carcinoma penis as compared to literate patients [48].

This study highlights the impact of education and rural dwelling on the clinical presentation, treatment pattern, and outcomes in carcinoma penis. There is a scarcity of published literature in this area and thus our results advocate the need to raise awareness about carcinoma penis in rural areas. However, the study has inherent biases associated with the small single-centre retrospective studies. Due to the absence of data on household income, occupation, and other determinants, we were unable to assess the impact of all socioeconomic factors on the outcomes.

## 10. Conclusions

Carcinoma penis is diagnosed a decade earlier in India and patients have more advanced disease at presentation. This may be attributable to low education status and rural habitation. Nodal involvement and the presence of metastatic disease at diagnosis are independent predictors of overall survival.

## Figures and Tables

**Table 1:** Baseline clinical characteristics of patients with carcinoma penis.

Characteristics	*n* = 102 (%)
**Median age (IQR) in years**	55.5 (42–65)
**Median symptom duration (IQR) months**	6.0 (4–12)
**Risk factors**	
Smoking	28 (27.5)
Phimosis	26 (25.5)
Balanoposthitis	22 (21.6)
HIV	2 (1.9)
Circumcision	1 (0.9)
**Symptoms**	
Pain	58 (56.8)
Discharge	34 (33.3)
Urinary symptoms	37 (36.3)
**ECOG PS**	
0	4 (3.9)
1	67 (65.6)
2	26 (25.5)
3	2 (1.9)
4	3 (2.9)
**Tumor Characteristics**	
Ulcero-proliferative growth	67 (65.7)
Lump	20 (19.6)
Ulcer	15 (14.7)
**Part of penis involved**	
Glans	72 (70.6)
Shaft	30 (29.4)
**Inguinal lymph node (clinical)**	73 (71.6)
**Scrotal involvement**	8 (7.8)

ECOG PS: Eastern Cooperative Oncology Group Performance Status, IQR interquartile range. Data summarized in frequency (percentage) except age and duration of symptoms which are mentioned in the median (IQR).

**Table 2:** Baseline tumor characteristics of patients with carcinoma penis.

Tumor Characteristics	*n* = 102 (%)
**T stage**	
T1	25 (24.5)
T2	25 (24.5)
T3	37 (36.2)
T4	7 (6.9)
TX	8 (7.8)
**N stage**	
N0	48 (47.1)
N1	8 (7.8)
N2	23 (22.5)
N3	12 (11.8)
NX	11 (10.8)
**M Stage**	
M0	96 (94.1)
M1	4 (3.8)
Not available	2 (1.9)
**Stage**	
I	22 (21.6)
II	23 (22.5)
III	29 (28.4)
IV	15 (14.7)
Not available	13 (12.8)
**Lymphovascular invasion**	12/41 (29.3)
**Perineural invasion**	11/41 (26.8)
**Histology**	
Well differentiated SCC	30 (29.4)
Moderately differentiated SCC	62 (60.8)
Poorly differentiated SCC	7 (6.8)
Others (Adenocarcinoma, Basaloid, Sarcomatoid)	3 (2.9)

**Table 3:** Differences in baseline characteristics of patients with carcinoma penis.

Characteristics	Distance		Rural/Urban		Education	
	<100 km *n* = 50 (%)	≥100 km *n* = 52 (%)	Rural *n* = 60 (%)	Urban *n* = 42 (%)	≤Primary *n* = 58 (%)	>Primary *n* = 33 (%)
**Median age (IQR) in years**	58 (42–65)	54.5 (40.5–62)	55 (40.5–63.5)	56.5 (42–66)	57 (45–65)	46 (40–62)
**Duration of symptoms >6 months**	18 (36.0)	24 (46.1)	26 (43.3)	16 (38.0)	24 (41.3)	14 (42.4)
**Risk factors**						
Smoking	16 (33.0)	12 (23.0)	16 (26.6)	12 (28.5)	18 (31.0)	9 (27.2)
Phimosis	14 (28.0)	12 (23.0)	14 (23.3)	12 (28.5)	15 (25.8)	8 (24.2)
**ECOG PS**						
0–1	33 (66.0)	37 (71.1)	42 (70.0)	28 (66.6)	43 (74.1)	20 (60.6)
2–4	17 (34.0)	15 (28.8)	18 (30.0)	14 (33.3)	15 (25.8)	13 (39.3)
**Inguinal lymph node (clinical)**	38 (76.0)	35 (67.3)	43 (72.1)	30 (71.4)	40 (68.9)	26 (78.7)
**T stage**						
T1	9 (18.0)	16 (30.7)	15 (25.0)	10 (23.8)	19 (32.7)	4 (12.1)
T2	12 (24.0)	13 (25.0)	15 (25.0)	10 (23.8)	13 (22.4)	9 (27.2)
T3	21 (42.0)	16 (30.7)	21 (35.0)	16 (38.0)	17 (29.3)	17 (51.5)
T4	1 (2.0)	6 (11.5)	7 (10.1)	0	6 (10.3)	1 (1.7)
**N stage**						
N0	22 (44.0)	26 (50.0)	25 (41.6)	23 (54.7)	26 (44.8)	17 (51.5)
N+	20 (40.0)	23 (44.2)	30 (50.0)	13 (30.9)	29 (50.0)	11 (18.9)
**M Stage**						
M1	1 (2.0)	3 (5.7)	4 (6.6)	0	3 (5.1)	1 (1.7)
**Stage**						
I	7 (14.0)	15 (28.8)	13 (21.6)	9 (21.4)	16 (27.5)	4 (12.1)
II	14 (28.0)	9 (17.3)	10 (10.6)	13 (30.9)	8 (13.7)	12 (36.3)
III	13 (26.0)	16 (30.7)	19 (31.6)	10 (23.8)	20 (34.4)	8 (24.2)
IV	6 (12.0)	9 (17.3)	13 (21.6)	2 (4.7)	10 (17.2)	4 (12.1)
**Lymphovascular invasion**	8/18 (44.4)	4/23 (16.6)	6/25 (24.0)	6/16 (37.5)	5/20 (25.0)	7/21 (33.3)
**Perineural invasion**	6/18 (33.3)	5/24 (20.8)	6/26 (23.0)	5/15 (33.3)	4/18 (22.2)	6/20 (30.0)
**Histology**						
WDSCC	15 (30.0)	15 (28.8)	16 (26.6)	14 (33.3)	17 (29.3)	9 (27.2)
MDSCC	29 (58.0)	33 (63.4)	39 (65.0)	23 (54.7)	35 (60.3)	23 (69.6)
PDSCC	5 (10.0)	2 (3.8)	3 (5.0)	4 (9.5)	4 (6.8)	1 (1.7)
Other histology *	1 (2.0)	2 (3.8)	2 (3.3)	1 (2.3)	2 (3.4)	0

ECOG PS Eastern Cooperative Oncology Group Performance Status, IQR interquartile range, MDSCC Moderately differentiated squamous cell carcinoma, PDSCC Poorly differentiated squamous cell carcinoma, WDSCC Well-differentiated squamous cell carcinoma. * Others include Adenocarcinoma, Basaloid, and Sarcomatoid histology. Data is summarized in frequency (percentage) except age which is mentioned in the median (IQR).

**Table 4:** Predictors of RFS and OS in carcinoma penis.

Parameter		Relapse Free Survival		Overall Survival	
		Hazard Ratio (CI)	*p* Value	Hazard Ratio (CI)	*p* Value
**Univariate analysis**					
T stage	T2	3.7 (1.1–11.6)	**0.0012**	2.4 (0.7–8.0)	**0.007**
	T3	5.5 (1.7–17.0)		6.1 (2.0–18.3)	
	T4	42.3 (4.1–428.5)		5.2 (1.2–21.3)	
LN positive		2.4 (1.2–5.0)	**0.01**	3.3 (1.1–6.6)	**0.0003**
Stage	II	4.2 (1.1–15.3)	**0.005**	11.0 (1.3–28.0)	**0.0002**
	III	6.4 (1.7–23.5)		18.7 (1.9–38.9)	
	IV	10.7 (1.8–72.5)		25.4 (2.2–86.1)	
ECOG PS > 1		2.9 (1.5–5.7)	**0.002**	2.1 (1.1–3.8)	**0.023**
Albumin		0.5 (0.3–0.9)	**0.04**	0.5 (0.3–0.8)	**0.01**
PNI		1.1 (0.78–1.57)	0.62	3.6 (1.2–10.7)	**0.022**
LVI		1.0 (0.76–1.5)	0.66	2.9 (0.98–8.7)	**0.054**
Metastatic disease		Not applicable		3.2 (1.0–10.7)	**0.049**
Distance		0.65 (0.35–1.2)	0.18	0.60 (0.333–1.1)	0.10
Education		0.97 (0.50–1.9)	0.94	1.00 (0.53–1.8)	0.98
Rural/urban		1.18 (0.63–2.2)	0.59	1.02 (0.55–1.87)	0.94
**Multivariate analysis**					
T stage		1.33 (0.55–3.2)	0.52	1.19 (0.61–2.3)	0.60
LN positive		1.13 (0.22–5.6)	0.87	3.58 (1.52–8.41)	**0.003**
Stage		1.75 (1.07–2.85)	**0.02**	1.82 (0.67–4.9)	0.23
ECOG PS > 1		0.81 (0.26–2.55)	0.75	1.06 (0.45–2.5)	0.87
Albumin		0.57 (0.28–1.17)	0.10	0.51 (0.25–1.06)	0.075
PNI		0.86 (0.28–2.6)	0.74	0.78 (0.21–2.8)	0.70
LVI		0.94 (0.29–3.0)	0.42	1.26 (0.32–4.9)	0.73
Metastatic disease		Not applicable		3.55 (1.02–12.34)	**0.045**
Distance		0.53 (0.12–2.2)	0.40	0.34 (0.09–1.2)	0.09
Education		0.67 (0.24–1.8)	0.44	1.7 (0.68–4.6)	0.23
Rural/urban		0.56 (0.11–2.7)	0.47	0.50 (0.12–2.0)	0.33

CI confidence interval, ECOG PS: Eastern Cooperative Oncology Group Performance Status, LVI lymphovascular invasion, OS overall survival, PNI perineural invasion, RFS relapse-free survival. Statistically significant values highlighted in bold.

## Data Availability

The datasets used and/or analysed during the current study are available from the corresponding author upon reasonable request.

## Supplementary Materials

The following supporting information can be downloaded at: https://www.mdpi.com/article/10.3390/curroncol30050345/s1, Table S1: Summary of management of patients treated from other centre; Table S2: Summary of initial management of patients with carcinoma penis.
