# Supplementary material for: Clinical Profile and Predictors of Survival in Carcinoma Penis Patients
Source: Curr Oncol. 2023 Apr 28;30(5):4563–74. doi: 10.3390/curroncol30050345 (PMC10216912; doi:10.3390/curroncol30050345)
Supplement: Supplementary file 1 [file curroncol-30-00345-s001.zip › curroncol-2302085-supplementary.pdf]

Supplementary Table S1. Summary of management of patients treated from other centers.

Initial Treatment	n = 45 (%)	Status at presentation	n = 45 (%)	Treatment at our center	n = 45
Partial penectomy	24 (53.3)	No Relapse	16 (35.5)	ILND +/- RT<br>Adjuvant RT<br>Observation	11<br>03<br>02
		Relapse Local	06 (13.3)	Palliative RT<br>Palliative Chemotherapy + RT	04<br>02
		Relapse distant + Local	02 (4.4)	Palliative Chemotherapy + RT<br>BSC	01<br>01
Partial penectomy + ILND	04 (8.8)	No Relapse	01 (2.2)	Adjuvant RT	01
		Relapse Local	02 (4.4)	Palliative RT	02
		Relapse distant	01 (2.2)	Palliative Chemotherapy	01
Partial penectomy + Adjuvant RT	02 (4.4)	No Relapse	00		
		Relapse distant	02 (4.4)	Palliative Chemotherapy	02
Total Penectomy	02 (4.4)	No Relapse	01 (2.2)	Observation	01
		Relapse distant	01 (2.2)	Palliative Chemotherapy	01
Circumcision	12 (26.6)	No Relapse	08 (17.8)	Partial penectomy + ILND + RT<br>Brachytherapy + EBRT<br>Brachytherapy<br>Observation	05<br>01<br>01<br>01
		Relapse distant	04 (8.8)	Palliative RT<br>Palliative Chemotherapy<br>BSC	02<br>01<br>01
Palliative Chemotherapy	01 (2.2)	Advanced disease	01 (2.2)	Palliative Chemotherapy	01

Supplementary Table S2. Summary of initial management of patients with carcinoma penis.

**Initial Treatment**	**n = 56 (%)**
Surgery	43 (76.8)
Partial penectomy +/- ILND	38 (67.8)
Total Penectomy +/- ILND	05 (9.0)
Brachytherapy + EBRT	02 (3.6)
Palliative treatment	10 (17.8)
Best supportive care	01 (1.8)

EBRT External beam radiation therapy, ILND Inguinal lymph node dissection
